# Bis(6-Diphenylphosphinoacenaphth-5-yl)Telluride as a Ligand toward Manganese and Rhenium Carbonyls

**DOI:** 10.3390/molecules23112805

**Published:** 2018-10-29

**Authors:** Truong Giang Do, Emanuel Hupf, Enno Lork, Jens Beckmann

**Affiliations:** Institut für Anorganische Chemie und Kristallographie, Universität Bremen, Leobener Straße 7, 28359 Bremen, Germany; do_giang@yahoo.de (T.G.D.); hupf@uni-bremen.de (E.H.); enno.lork@uni-bremen.de (E.L.)

**Keywords:** ligand design, transition metal complex, tellurium, manganese, rhenium

## Abstract

The reaction of the previously known bis(6-diphenylphosphinoacenaphthyl-5-)telluride (6-Ph_2_P-Ace-5-)_2_Te (**IV**) with (CO)_5_ReCl and (CO)_5_MnBr proceeded with the liberation of CO and provided *fac*-(6-Ph_2_P-Ace-5-)_2_TeM(X)(CO)_3_ (*fac*-**1**: M = Re, X = Cl; *fac*-**2**: M = Mn, X = Br), in which **IV** acts as bidentate ligand. In solution, *fac*-**1** and *fac*-**2** are engaged in a reversible equilibrium with *mer*-(6-Ph_2_P-Ace-5-)_2_TeM(X)(CO)_3_ (*mer*-**1**: M = Re, X = Cl; *mer*-**2**: M = Mn, X = Br). Unlike *fac*-**1**, *fac*-**2** is prone to release another equivalent of CO to give (6-Ph_2_P-Ace-5-)_2_TeMn(Br)(CO)_2_ (**3**), in which **IV** serves as tridentate ligand.

## 1. Introduction

**Scheme 1:**
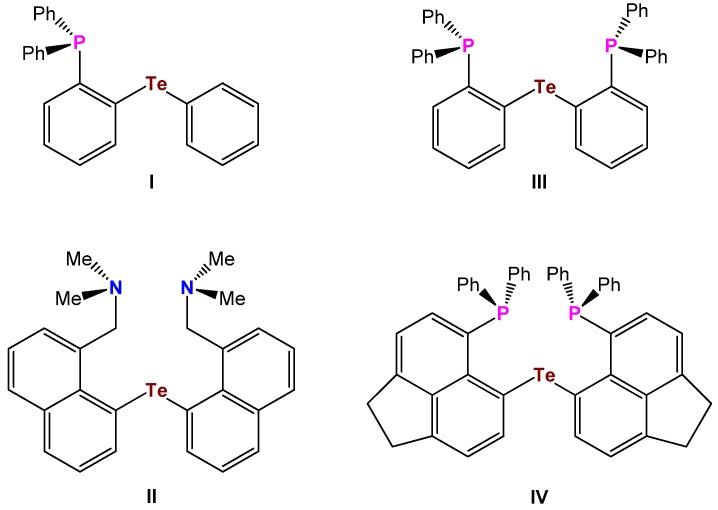
Bidentate and tridentate telluroether ligands **I**–**IV**.

Transition metal complexes composed of multidentate ligands based upon tellurium are far less explored than those of sulfur and selenium, which might be due to the lack of easily available ligands containing tellurium, as well as the historical misconception that organotellurium compounds were extremely malodorous and toxic [1,2,3,4,5,6,7,8]. Up to the 1970s, studies of coordination chemistry were mostly restricted to monodentate ligands. The first bidentate telluroether ligand **I** (Te, P-type) was described by Gysling and Luss in 1984 [9], whereas the first tridentate telluroethers **II** (N,Te,N-type), and **III** (P,Te,P-type) were reported by Singh et al. [10] as well as Lin and Gabbaï (Scheme 1) [11].

In preceding work, we reported on the synthesis of bis(6-diphenylphosphino­acenaphth-5-yl)telluride (**IV**) [12], which also holds potential as a tridentate telluroether ligand. Herein, we describe the reaction of **IV** with (CO)_5_MnBr and (CO)_5_ReCl, giving rise to the formation of three octahedral transition metal carbonyl complexes with this ligand.

## 2. Results and Discussion

**Scheme 2:**
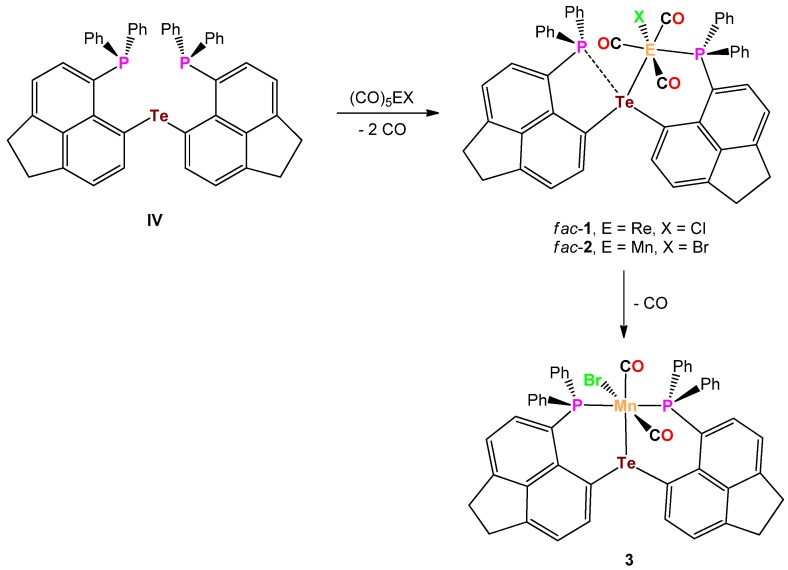
Synthesis of the complexes *fac*-**1**, *fac*-**2**, and **3**.

The reaction of bis(6-diphenylphosphinoacenaphth-5-yl)telluride (6-Ph_2_P-Ace-5-)_2_Te (**IV**) with (CO)_5_ReCl in THF at 65° for 7 days afforded an 1:1 isomeric mixture of the complex *fac/mer*-(6-Ph_2_P-Ace-5-)_2_TeRe(Cl)(CO)_3_ (*fac*-**1/***mer*-**1**), which slowly interconvert in solution. The progress of the reaction was followed by ^31^P NMR spectroscopy. Upon cooling to r.t. solely *fac*-**1** precipitates from the reaction mixture and was isolated in 45% isolated yield (Scheme 2).

**Scheme 3:**
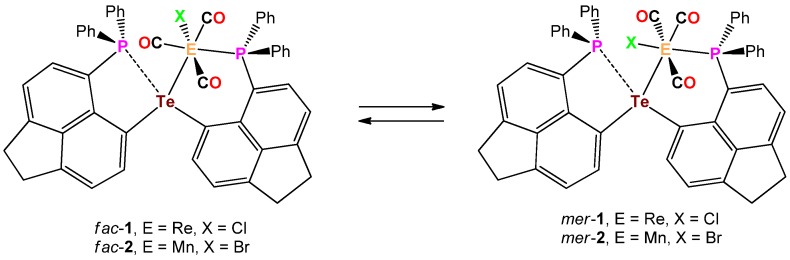
Dynamic equilibrium between *fac*-**1** and *mer*-**1** as well as *fac*-**2** and *mer*-**2**.

**Figure 1:**
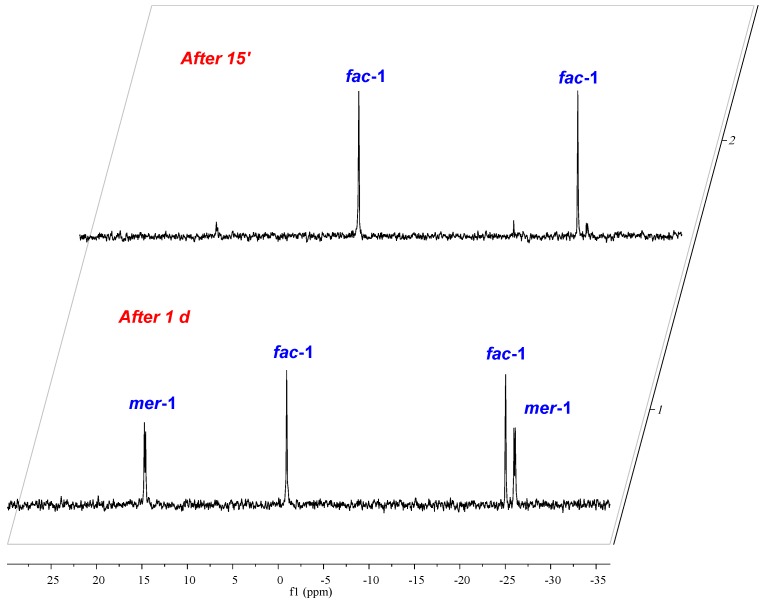
Equilibration between *fac*-**1** and *mer*-**1** in the CDCl_3_ solution.

The analogous reaction of **IV** with (CO)_5_MnBr in THF at 65° occurred within 32 h and gave rise to the isomeric mixture of *fac*/*mer*-(6-Ph_2_P-Ace-5-)_2_TeMe(Br)(CO)_3_ (*fac*-**2**/*mer*-**2**) and as was already observed for the Te-Re complex *fac*-**1**, *fac*-**2** precipitates solely from the reaction mixture upon cooling to r.t. and was isolated in 47% yield (Scheme 2). In both complexes *fac*-**1** and *fac*-**2**, the telluroether **IV** serves as bidentate ligand. In the solid-state, *fac*-**1** and *fac*-**2** are reasonably stable toward moist air and show no signs of decomposition even after prolonged times of storage. Unfortunately, both complexes show only poor solubility in the most common solvents used for NMR spectroscopy. In CDCl_3_ solution, both *fac*-**1** and *fac*-**2** are in equilibrium with their related *meridional* complexes *mer*-**1** and *mer*-**2**, which can be inferred by ^31^P-NMR spectroscopy (Scheme 3). The ^31^P-NMR spectrum of a freshly prepared solution of *fac*-**1** in CDCl_3_, shows almost exclusively two chemical shifts at δ = −0.9 and −25.0 ppm of equal intensity, which are assigned to the P atom that coordinates to the Re atom and the P atom that engages in interaction with the Te atom (the assignment is based upon the comparison with *fac*-[Re(Cl)(CO)_3_(PPh_2_C_10_H_6_PPh_2_)] [13], ([(Ph_2_P(Me_2_pz)_2_)Re(CO)_3_Br] and [(Ph_2_P(Me_2_pz))Re(CO)_4_Br (pz = pyrazole) [14]). However, the formation of *mer*-**1** can already be detected even from the freshly prepared solution of *fac*-**1**, showing chemical shifts at δ = 14.7 and −26.4 ppm, which increase in intensity over time (Figure 1). It should be noted that the chemical shifts of *fac*-**1** consist of singlets, whereas the doublets were observed for *mer*-**1** with a coupling constant of *J*(^31^P-^31^P) = 11.6 Hz. In contrast, the Te–Mn complex **2** shows significantly faster formation of *mer*-**2** (δ (^31^P) = 50.0 and −28.0 ppm) from a freshly prepared solution of *fac*-**1** (δ (^31^P) = 36.2 and −25.6 ppm, Figure S2d).

When the reaction of **IV** with (CO)_5_MnBr was repeated with a longer reaction time of 7 days, a different product, namely (6-Ph_2_P-Ace-5-)_2_TeMn(Br)(CO)_2_ (**3**) formed, which was obtained in 40% isolated yield (Scheme 2). The formation of **3** can be rationalized by the liberation of CO from *fac*-**2** and/or *mer*-**2**. In **3**, the telluroether serves as tridentate ligand. Notably, a similar reactivity of *fac*-**1** was not observed. The solubility of **3** in the most common solvents is slightly higher than those of *fac*-**1** and *fac*-**2**, which allowed the acquisition of full set of NMR data. The ^31^P-NMR spectrum (CDCl_3_) of **3** shows a broad signal at *δ* = 59.7 ppm (ω_1/2_ = 110 Hz), whereas the ^125^Te-NMR spectrum exhibits a singlet at *δ* = 753.1 pm that is slightly high-field shifted in comparison to **IV** (*δ* = 704.4 ppm) [12].

**Figure 2:**
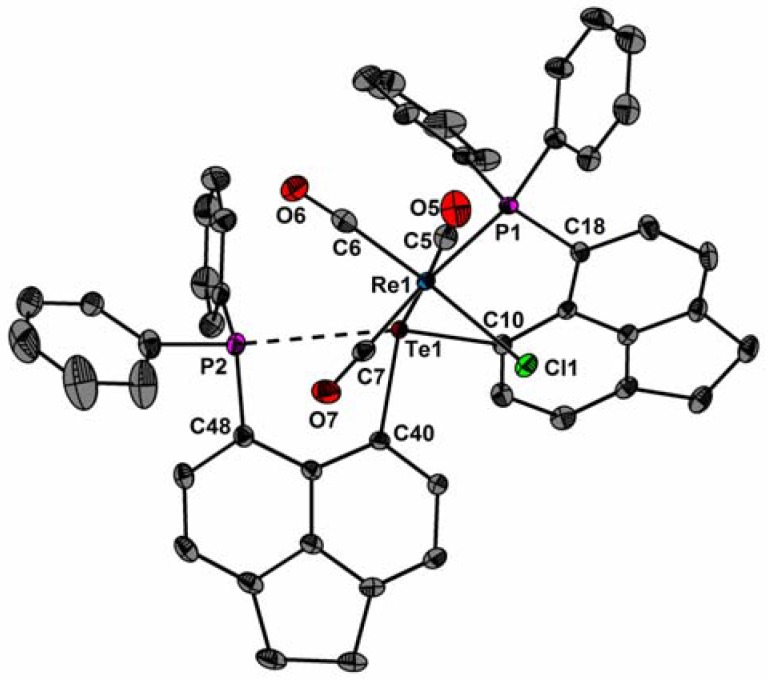
Molecular structure of *fac*-**1** showing 50% probability ellipsoids and the crystallographic numbering scheme.

**Figure 3:**
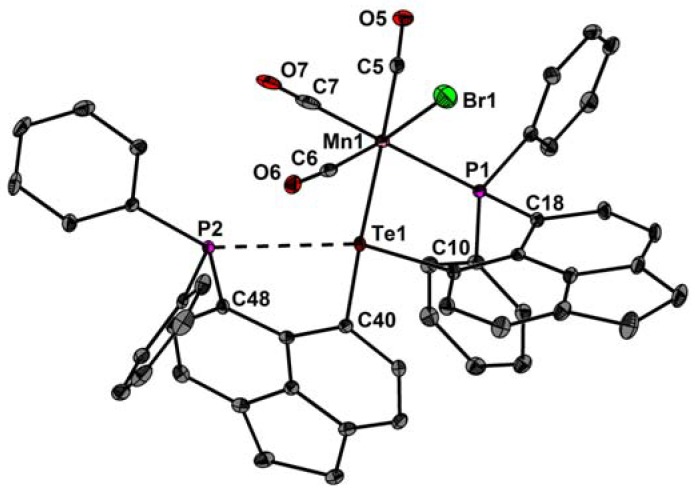
Molecular structure of *fac*-**2** showing 50% probability ellipsoids and the crystallographic numbering scheme.

**Figure 4:**
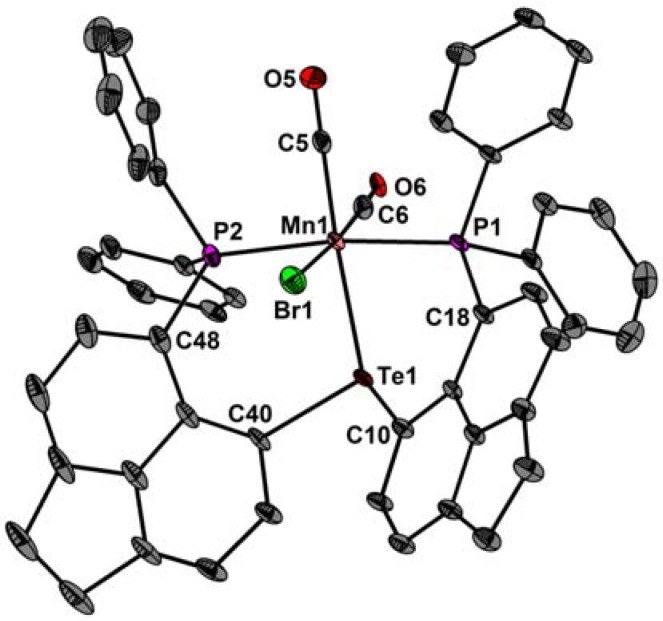
Molecular structure of **3** showing 50% probability ellipsoids and the crystallographic numbering scheme.

The molecular structures of *fac*-**1**, *fac*-**2**, and **3** are shown in Figure 2, Figure 3 and Figure 4. Selected bond parameters are collected in Table 1. The spatial arrangement of the Re and Mn atoms is octahedral. In *fac*-**1** and *fac*-**2**, only one P atom coordinates to the Re and Mn atoms, whereas the other P atoms engage in a through-space interactions with the Te atoms. Consistent with -the covalent radii of Mn and Re, the Mn–Te bond length of *fac*-**2** (2.599(1) Å) and **3** (2.546(1) Å) are smaller than the Re–Te bond length of *fac*-**1** (2.733(1) Å). The Mn–Te distances are in between values reported for [Mn(CO)_4_TePh]_2_ (mean 2.674(2) Å) [15,16,17] and [Cp′(CO)_2_Mn]_2_TeMes^+^ (2.44 Å) [18].

Likewise, the Mn–P bond length of *fac*-**2** (2.343(1) Å) and **3** (2.341(1) Å) is smaller than the Re–P bond length of **1** (2.466(1) Å). The average Mn–P distances are comparable with those of [Mn(Cl)(CO)_3_(κ^1^-PPh_2_C_10_H_6_PPh_2_)] (2.328(1) Å) [13] and *^i^*^Pr^PN^H^PMn(CO)_2_Br (2.263 (1) Å) [19]. The Re(1)–C(5)/C(6) bond distances of *fac*-**1** (2 × 1.930(2) Å) and the Mn(1)–C(5)/C(6) bond distances of *fac*-**2** (1.817(6), 1.819(5) Å) are very similar to those of [ReCl(CO)_3_(PPh_2_C_10_H_6_PPh_2_)] (mean 1.952(16) Å)[13] and *fac*-[MnCl(CO)_3_(PPh_2_C_10_H_6_PPh_2_)] (mean 1.815(2) Å) [20]. Due to the *trans*-effect of the P atom, the Re(1)–C(7) bond distance of *fac*-**1** (1.951(2) Å) and the Mn(1)–C(7) distance of *fac*-**2** (1.915(7) Å) are slightly longer. The Mn(1)–C(5) and Mn(1)–C(6) bond lengths of **3** (1.800(4), 1.811(5) Å) are in average very similar than that of *fac*-[Mn(CO)_3_(dppbz)Br] (mean 1.807(4) Å; dppbz = 1,2-(diphenylphosphino)benzene) [21]. In the carbonyl region of the IR spectrum of *fac*-**1** three strong absorption bands at $\tilde{v}$ = 2018, 1928, and 1882 cm^−1^ are visible, respectively, consistent with a facial arrangement of three carbonyl groups [12,22]. The IR spectrum of **3** reveals asymmetric and symmetric stretching vibrations at $\tilde{v}$ = 1936 and 1852 cm^−1^, which compare well with those of (^i^PrPONOP)Mn(CO)_2_Br (1866, 1946 cm^−1^) and ^i^PrPNHPMn(CO)_2_Br (1824, 1916 cm^−1^) [19].

## 3. Conclusions

The present study demonstrates the ability of bis(6-diphenylphosphinoacenaphthyl-5-)telluride (6-Ph_2_P-Ace-5-)_2_Te (**IV**) [12] to act as a bidentate and tridentate ligand toward transition metals carbonyls. The reaction of **IV** with (CO)_5_ReCl and (CO)_5_MnBr provided three complexes, namely *fac*-(6-Ph_2_P-Ace-5-)_2_TeM(X)(CO)_3_ (*fac*-**1**: M = Re, X = Cl; *fac*-**2**: M = Mn, X = Br) and (6-Ph_2_P-Ace-5-)_2_TeMn(Br)(CO)_2_ (**3**). In solution, the facial complexes, *fac*-**1** and *fac*-**2**, are engaged in a reversible equilibrium with their meridional complexes, *mer*-(6-Ph_2_P-Ace-5-)_2_TeM(X)(CO)_3_ (*mer*-**1**: M = Re, X = Cl; *mer*-**2**: M = Mn, X = Br).

## 4. Experimental Section

*General.* Reagents were obtained commercially (Sigma-Aldrich, Taufkirchen, Germany) and were used as received. Dry solvents were collected from an SPS800 mBraun solvent system. Bis(6-diphenylacenaphth-5-yl)telluride, (6-Ph_2_P-Ace-5-)_2_Te (**IV**) was prepared according to literature procedures [12]. ^1^H-, ^13^C-, ^31^P-, and ^125^Te-NMR spectra were recorded at room temperature using a Bruker Avance-360 and a Bruker Avance-200 spectrometer and are referenced to tetramethylsilane (^1^H, ^13^C), phosphoric acid (85% in water) (^31^P), and dimethyltelluride (^125^Te). Chemical shifts are reported in parts per million (ppm), and coupling constants (*J*) are given in Hertz (Hz). The FTIR spectra were recorded on Thermo Scientific Nicolet^TM^ iS10. The ESI-MS spectra were obtained with a Bruker Esquire-LC MS. Dichloromethane/acetonitrile solutions (c = 1 × 10^−6^ mol L^−1^) were injected directly into the spectrometer at a flow rate of 3 µL min^−1^. Nitrogen was used both as a drying gas and for nebulization with flow rates of approximately 5 L min^−1^ and a pressure of 5 psi. Pressure in the mass analyzer region was usually about 1 × 10^−5^ mbar. Spectra were collected for 1 min and averaged. The nozzle-skimmer voltage was adjusted individually for each measurement.

*Synthesis of (6-Ph_2_P-Ace-5-)_2_TeRe(CO)_3_Cl* (*fac*-**1**): Re(CO)_5_Cl (9.20 mg, 25.4 µmol) was added to a solution of (6-Ph_2_P-Ace-5-)_2_Te (20.0 mg, 24.9 μmol) in THF (5 mL). The mixture was continually stirred at 65 °C for 7 d. The product precipitated upon cooling to room temperature as colorless microcrystals. (12.5 mg, 11.3 µmol, 45%; Mp. 197 °C (dec.)). Due to the low-solubility and the equilibrium of *fac*-**1** and *mer*-**1** in solution, reliable ^13^C- and ^125^Te-NMR spectra could not be obtained.

*fac*-**1**: ^1^H-NMR (200.1 MHz, CDCl_3_) *δ* = 7.99 (d, ^3^*J* = 7.9 Hz, 1H), 7.42 (m, 18H), 7.18 (dd, *J* = 7.9, 3.4 Hz, 4H), 6.71 (d, ^3^*J* = 7.4 Hz, 1H), 6.22 (d, ^3^*J* = 7.5 Hz, 1H), 3.50 (s, 4H), 3.45–3.15 (m, 4H) ppm. ^31^P-NMR (81.0 MHz, CDCl_3_) *δ* = −0.9 (s), −25.0 (s) ppm. ESI-MS (CH_2_Cl_2_/CH_3_CN 1:10, positive mode): *m*/*z* = 1073.4 (C_51_H_38_O_3_P_2_ReTe) for [(6-Ph_2_P-Ace-5-)_2_Te-Re(CO)_3_]^+^. FTIR: $\tilde{v}$ (CO) = 2018, 1928, 1882 cm^−1^. *mer*-**1**: ^31^P-NMR (81.0 MHz, CDCl_3_) *δ* = 14.7 (d, *J*(^31^P-^31^P) = 11.6 Hz), −26.4 (d, *J*(^31^P-^31^P) = 11.6 Hz) ppm.

*Synthesis of (6-Ph_2_P-Ace-5-)_2_TeMn(CO)_3_Br* (*fac*-**2**): Mn(CO)_5_Br (17.5 mg, 64.4 μmol) was added to a solution of (6-Ph_2_P-Ace-5-)_2_Te (50 mg, 62.3 μmol) in THF (10 mL). The mixture was continually stirred at 65 °C for 32 h. The product precipitated upon cooling to room temperature as orange microcrystals (30 mg, 29.4 μmol, 47%; Mp. 185 °C (dec.)). Due to the low-solubility, the equilibrium between *fac*-**2** and *mer*-**2** in solution and the concomitant formation of **3** reliable ^13^C- and ^125^Te-NMR spectra could not be obtained.

*fac*-2: ^31^P-NMR (81.0 MHz, CDCl_3_) *δ* = 50.4 (s, br), 39.9 (s, br), −27.4 (d, *J*(^31^P-^31^P) = 7.2 Hz) ppm. *mer*-2: ^1^H-NMR (200.1 MHz, CDCl_3_) *δ* = 7.95 (d, ^3^*J* = 7.2 Hz, 2H), 7.80–6.85 (m, 26H), 3.41 (m, 8H) ppm. ^31^P-NMR (81.0 MHz, CDCl_3_) *δ* = 50.0 (s, br), −27.9 (d, *J*(^31^P-^31^P) = 7.2 Hz) ppm. ESI-MS (CH_2_Cl_2_/CH_3_CN 1:10, positive mode): *m*/*z* = 943.1 (C_51_H_38_O_3_P_2_MnTe) for [(6-Ph_2_P-Ace-5-)_2_Te–Mn(CO)_3_]^+^. FTIR: $\tilde{v}$ (CO) = 2011, 1939, 1895 cm^−1^.

Synthesis of (6-Ph_2_P-Ace-5-)_2_TeMn(CO)_2_Br (3). Mn(CO)_5_Br (7.00 mg, 25.5 μmol) was added to a solution of (6-Ph_2_P-Ace-5-)_2_Te (20.0 mg, 24.9 μmol) in THF (5 mL). The mixture was continually stirred at 65 °C for 7 d. The product precipitated upon cooling to room temperature as yellow microcrystals (10.0 mg, 10.0 μmol, 40%; Mp. 150 °C (dec.)).

^1^H-NMR (360.3 MHz, CD_2_Cl_2_) *δ* = 8.03 (s, 4H, H_o_), 7.60 (d, *J* = 7.1 Hz, 2H, H_4_), 7.52 (s, 6H, H_m+p_), 7.35–7.19 (m, 8H, H_m’+p’_+ H_8_), 7.19–7.05 (m, 6H, H_o’_+ H_4_), 7.00 (m, 2H, H_7_), 3.50–3.34 (m, 8H, H_1+2_). ^13^C-NMR (90.6 MHz, CD_2_Cl_2_) *δ* = 150.8 (s, C_c_), 150.2 (s, C_d_), 139.9 (t, *J* = 4.0 Hz, C_b_), 139.0 (s, C_4_), 136.8 (t, *J* = 5.1 Hz, C_a_), 136.2 (s, C_7_), 135.7 (t, *J* = 18.1 Hz, C_i_), 135.7 (d, *J* = 18.0 Hz, C_i’_), 134.5 (t, *J* = 4.9 Hz, C_o_), 133.1 (t, *J* = 4.6 Hz, C_o’_), 129.8 (s, C_m_), 129.0 (s, C_m’_), 127.8 (t, *J* = 4.4 Hz, C_p_), 127.5 (t, *J* = 4.2 Hz, C_p’_), 127.0 (d, *J* = 17.5 Hz, C_6_), 119.5 (t, *J* = 3.9 Hz, C_3_), 119.4 (s, C_8_), 104.6 (t, *J* = 7.0 Hz, C_5_), 29.8 (s, C_1_), 29.7 (s, C_2_). ^31^P-NMR (81.0 MHz, CD_2_Cl_2_) *δ* = 59.7 (s, br). ^125^Te-NMR (113.7 MHz, CD_2_Cl_2_) *δ* = 753.1 (s). ESI-MS (CH_2_Cl_2_/CH_3_CN 1:10, positive mode): *m*/*z* = 859.4 (C_50_H_36_MnO_2_P_2_Te) for [[(6-Ph_2_P-Ace-5-)_2_Te–Mn(CO)_2_]–2CO]^+^. FTIR: $\tilde{v}$ (CO) = 1936 (s), 1852 (s) cm^−1^.

X-ray crystallography. Single crystals of *fac*-**1**, *fac*-**2**, and **3** were grown by slow evaporation of CH_2_Cl_2_ solutions that contained small amounts of *n*-hexane. Intensity data of *fac*-**1**, *fac*-**2**·CH_2_Cl_2_, and **3**·2 CH_2_Cl_2_ were collected at 100 K on a Bruker Venture D8 diffractometer with graphite-monochromated Mo-Kα (0.7107 Å) radiation. All structures were solved by direct methods and refined based on F^2^ by use of the SHELX program package as implemented in WinGX [23]. Strongly disordered solvent molecules were accounted using the SQUEEZE routine for **1** and **2** [24]. All non-hydrogen atoms were refined using anisotropic displacement parameters. Hydrogen atoms attached to carbon atoms were included in geometrically calculated positions using a riding model. Crystal and refinement data are collected in Table 2. 

Figure 1, Figure 2, Figure 3 and Figure 4 were created using DIAMOND [25]. Crystallographic data (excluding structure factors) for the structural analyses have been deposited with the Cambridge Crystallographic Data Centre. Copies of this information may be obtained free of charge from The Director, CCDC, 12 Union Road, Cambridge CB2 1EZ, UK (Fax: +44-1223-336033; e-mail: deposit@ccdc.cam.ac.uk).

## Figures and Tables

**Table 1:** Experimental interatomic distances [Å] and angles [°] of *fac*-**1**, *fac*-**2**, and **3**.

	*fac*-1 (M = Re)	*fac*-2 (M = Mn)	3 (M = Mn)
**Bond Lengths and Angles**			
M(1)–Te(1)	2.733(1)	2.599(1)	2.546(1)
M(1)–P(1)	2.466(1)	2.343(1)	2.293(1)
M(1)–P(2)	-	-	2.277(1)
M(1)–X(1)	2.487(1)	2.498(1)	2.540(1)
M(1)–C(5)	1.930(2)	1.819(5)	1.800(4)
M(1)–C(6)	1.930(2)	1.817(6)	1.811(4)
M(1)–C(7)	1.951(2)	1.915(7)	-
C(5)–O(5)	1.142(2)	1.127(7)	1.146(6)
C(6)–O(6)	1.121(2)	1.108(7)	1.037(5)
C(7)–O(7)	1.141(2)	0.993(8) ^a^	-
P(1)–M(1)–P(2)	-	-	170.8(1)
P(1)–M(1)–C(7)	171.9(1)	171.2(2)	-
X(1)–M(1)–C(6)	176.2(1)	173.9(2)	176.3(1)
Te(1)–M(1)–C(5)	174.0(1)	175.9(2)	175.6(1)
***peri* Region distances**			
P(1)···Te(1)	3.363(1)	3.466(1)	3.247(2)
P(1)···Te(2)	3.111(1)	3.141(1)	3.429(1)
***peri* Region bond Angles**			
P(1)–C(18)–C(19)/P(2)–C(48)–C(49)	122.9(1)/119.5(1)	124.6(3)/118.8(3)	124.3(3)/121.0(3)
C(10)–C(19)–C(18)/C(40)–C(49)–C(48)	130.2(1)/129.0(1)	129.8(4)/129.9(4)	130.3(4)/129.8(4)
Te(1)–C(10)–C(19)/Te(1)–C(40)–C(49)	127.6(1)/122.0(1)	129.6(3)/124.1(3)	123.2(3)/126.1(3)
∑ of bay angles	380.7(3)/370.5(3)	380.0(10)/372.8(1)	377.8(1)/376.9(1)
Splay angle ^b^	20.7(3)/10.5(3)	20(10)/12.8(1)	17.8(1)/17.4(1)
**Out-of-Plane Displacement**			
Te(1) ^c^	0.198(1)/−0.626(1)	−0.191(1)/−0.390(1)	0.0899(3)/−0.666(1)
P(1)	−0.203(1)	0.097(1)	−0.160(1)
P(2)	0.278(1)	0.263(1)	0.543(1)
**Central Acenaphthene Ring Torsion Angles**			
C:(13)–(14)–(19)–(18)/(43)–(44)–(49)–(48)	175.5(1)/177.9(1)	−179.3(6)/−173.3(5)	179.4(4)/169.4(5)
C:(15)–(14)–(19)–(10)/(45)–(44)–(49)–(40)	178.7(1)/176.2(1)	178.5(5)/−177.5(5)	179.4(4)/178.6(5)

^a^ This value might be erroneously low due to unresolved disorder. ^b^ Splay angle: sum of the three bay region angles −360°. ^c^ *fac*-**1**, *fac*-**2**, and **3** show a transoid out-of-plane displacement.

**Table 2:** Crystal data and structure refinement of *fac*-**1**, *fac*-**2**, and **3**.

	*fac*-1	*fac*-2·CH_2_Cl_2_	3·2 CH_2_Cl_2_
Formula	C_51_H_36_ClO_3_P_2_ReTe	C_52_H_38_BrCl_2_MnO_3_P_2_Te	C_52_H_40_BrCl_4_MnO_2_P_2_Te
Formula weight, g mol^−1^	1107.99	1106.11	1163.03
Crystal system	Triclinic	Monoclinic	Triclinic
Crystal size, mm	0.09 × 0.08 × 0.06	0.10 × 0.08 × 0.05	0.09 × 0.08 × 0.08
Space group	*P* $\bar{1}$	*P*2_1_/*n*	*P* $\bar{1}$
*a*, Å	12.2258(3)	10.2073(2)	12.542(5)
*b*, Å	14.3531(3)	22.4724(5)	12.738(5)
*c*, Å	14.8486(3)	19.7629(5)	15.516(5)
α, °	78.509(1)	90	100.490(5)
β, °	77.874(1)	101.421(1)	100.078(5)
γ, °	89.216(1)	90	100.930(5)
*V*, Å^3^	2495.33(9)	4443.5(2)	2336(2)
*Z*	2	4	2
ρ_calcd_, Mg m^−3^	1.475	1.653	1.653
μ (Mo *Kα*), mm^−1^	3.163	2.077	2.089
*F*(000)	1080	2200	1156
θ range, deg	2.31 to 29.57	2.29 to 27.56	2.38 to 30.12
Index ranges	−16 ≤ h ≤ 16	−13 ≤ h ≤ 12	−17 ≤ h ≤ 17
	−19 ≤ k ≤ 19	−29 ≤ k ≤ 29	−17 ≤ k ≤ 17
	−20 ≤ l ≤ 20	−25 ≤ l ≤ 25	−21 ≤ l ≤ 21
No. of reflns collected	275,626	120,248	202,873
Completeness to *θ*_max_	99.8%	99.8%	99.8%
No. indep. Reflns	13,983	10,262	13,719
No. obsd reflns with (*I* > 2*σ*(*I*))	13,702	8891	11,512
No. refined params	532	553	556
GooF (*F*^2^)	1.074	1.042	1.054
*R*_1_ (*F*) (*I* > 2*σ*(*I*))	0.0148	0.0559	0.0553
*wR*_2_ (*F*^2^) (all data)	0.0385	0.1690	0.1529
Largest diff peak/hole, e Å^−3^	0.844/−0.846	1.724/−1.561	1.955/−1.665
CCDC number	1861602	1861603	1861604

## Supplementary Materials

The following are available online.
