# Supplementary material for: Bis(6-Diphenylphosphinoacenaphth-5-yl)Telluride as a Ligand toward Manganese and Rhenium Carbonyls
Source: Molecules. 2018 Oct 29;23(11):2805. doi: 10.3390/molecules23112805 (PMC6278489; doi:10.3390/molecules23112805)
Supplement: Supplementary file 1 [file molecules-23-02805-s001.pdf]

Supporting Information

NMR spectra, IR spectra, crystal and refinement data and bond parameters of *fac*-**1**, *fac*-**2** and **3**. Crystallographic data (CIF).

Contents

Figure S1a ^{1}H-NMR spectrum of **1**.

Figure S1b ^{31}P{^{1}H}-NMR spectrum of **1**.

Figure S1c IR spectrum of **1**.

Figure S2a ^{31}P{^{1}H}-NMR spectrum of *fac*-**2** and *mer*-**2**.

Figure S2b IR spectrum of *fac*-**2** and *mer*-**2**.

Figure S2c ^{1}H-NMR spectrum of *mer*-**2**.

Figure S2d ^{31}P{^{1}H}-NMR spectrum of *mer*-**2**.

Figure S2e IR spectrum of *mer*-**2**.

Figure S3a ^{1}H-NMR spectrum of **3**.

Figure S3b ^{13}C{^{1}H}-NMR spectrum of **3**.

Figure S3c ^{31}P{^{1}H}-NMR spectrum of **3**.

Figure S3d ^{125}Te{^{1}H}-NMR spectrum of **3**.

Figure S3e IR spectrum of **3**.

Figure S4a Stacked plot of the ^{31}P{^{1}H}-NMR spectra showing formation and the equilibrium between *fac*-**2**, *mer*-**2** and the formation of **3**.

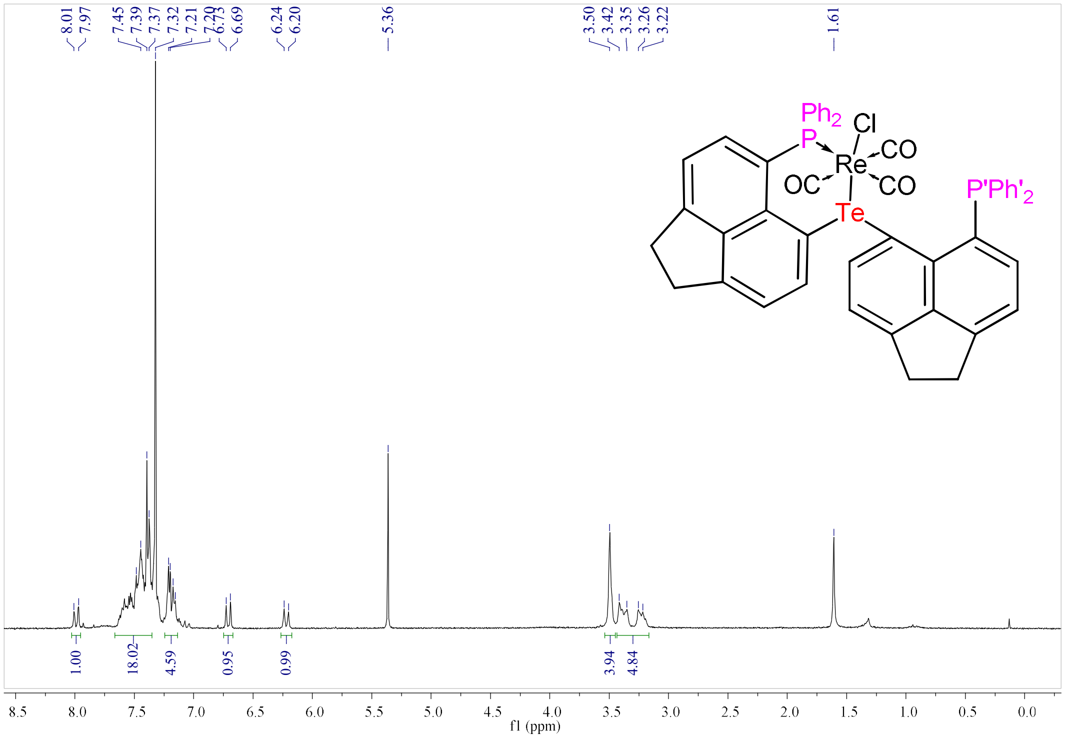

Figure S1a. ^{1}H-NMR spectrum of **1** (CD_2Cl_2).

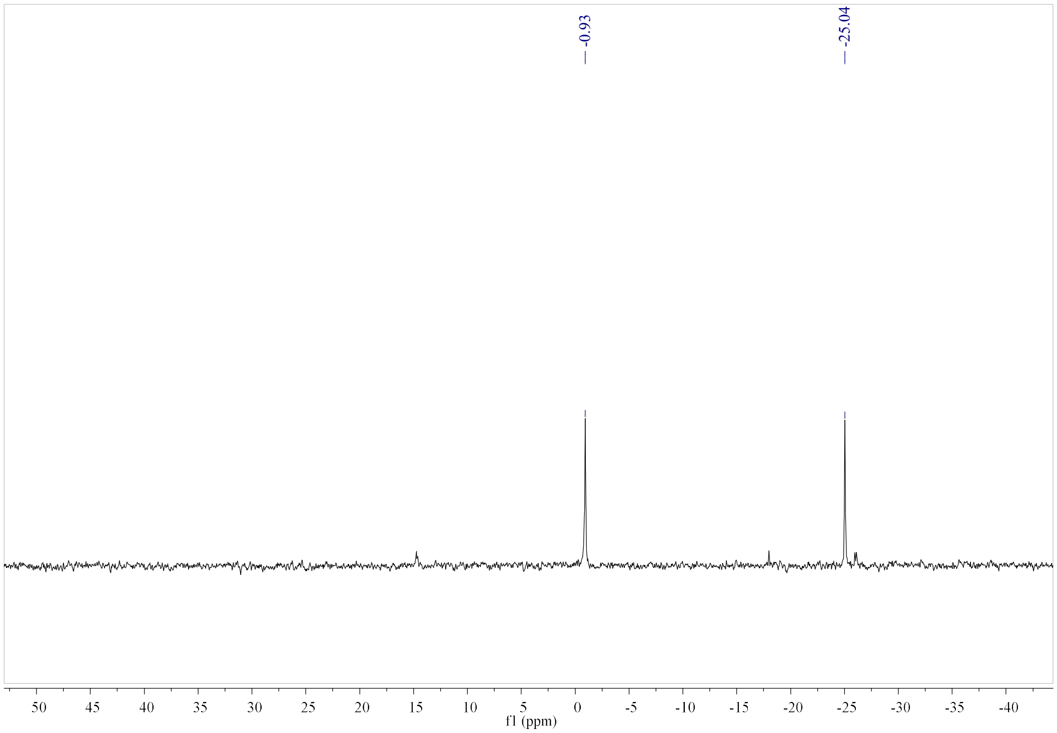

Figure S1b. ^{31}P{^{1}H}-NMR spectrum of **1** (CD_2Cl_2).

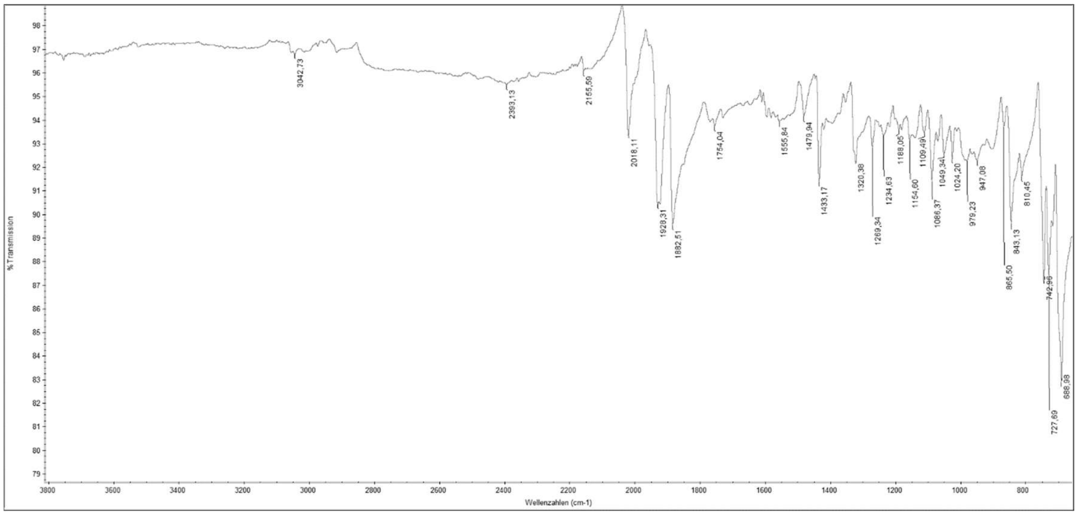

Figure S1c. IR spectrum of **1**.

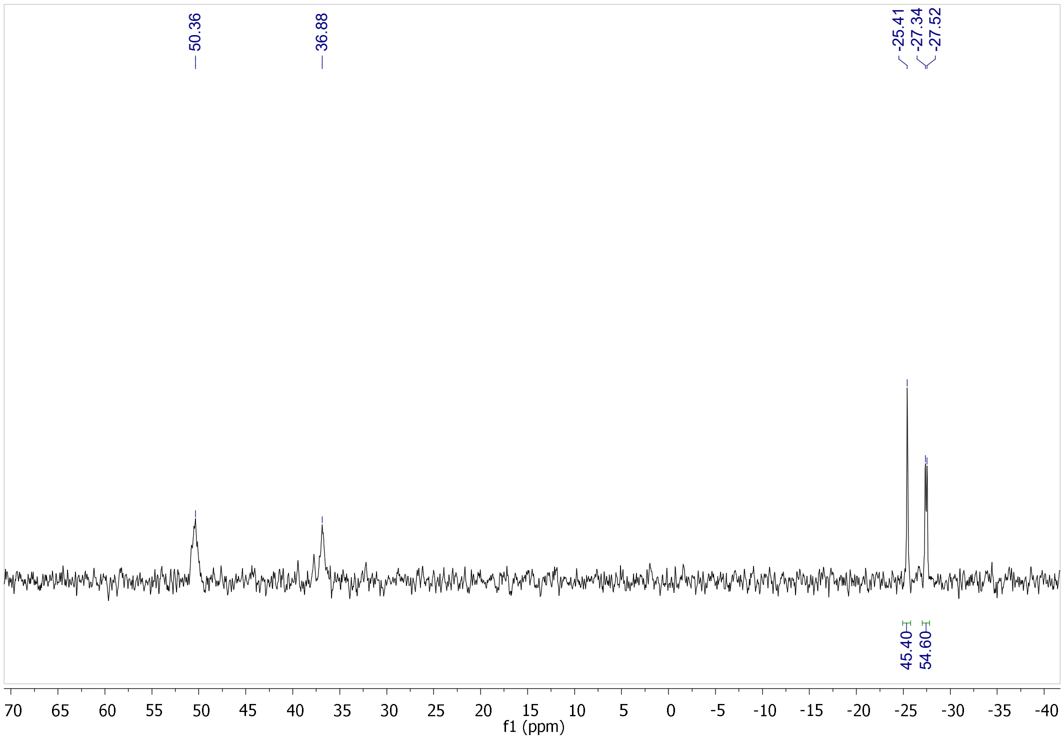

Figure S2a. ^{31}P-NMR of **2**(54% of *fac*-**2**/ 45% of *mer*-**2**) (CD_2Cl_2).

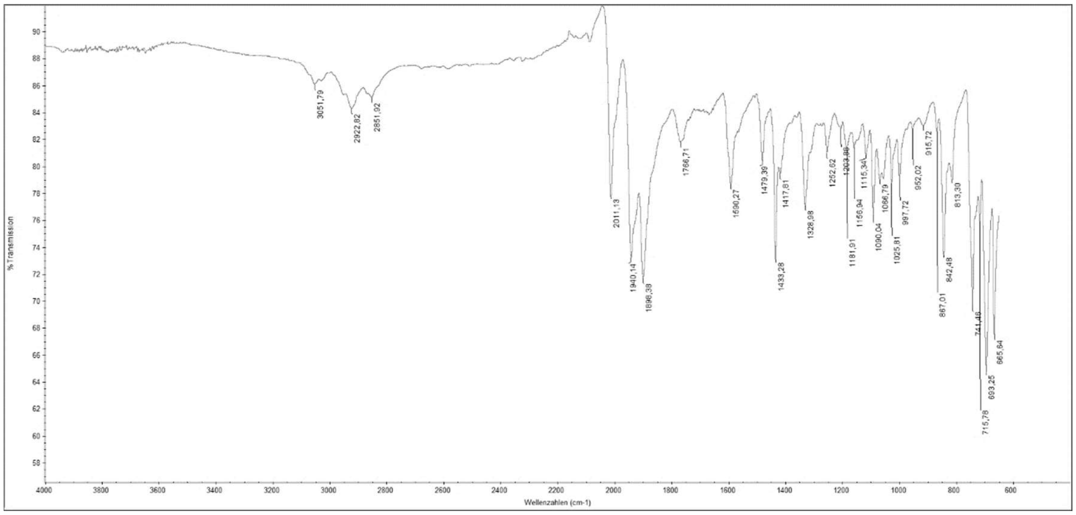

Figure S2b. IR spectrum of **2**(54% of *fac*-**2**/ 45% of *mer*-**2**).

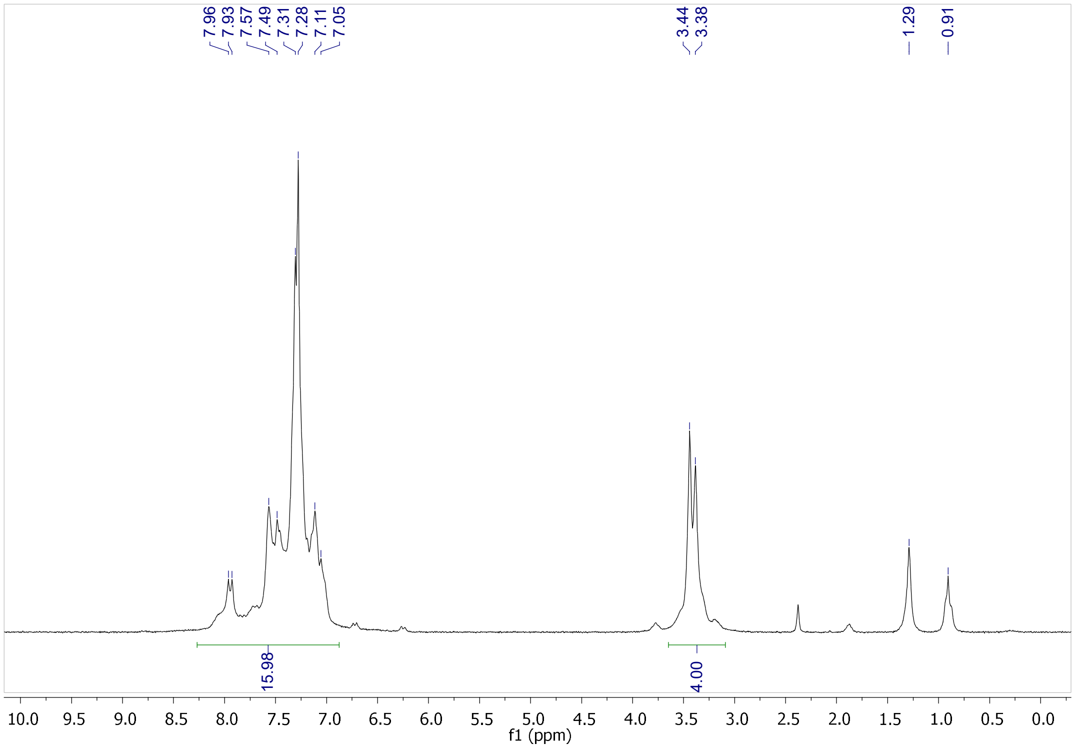

Figure S2c. ^{1}H-NMR spectrum of *mer*-**2** ($CDCl_3$).

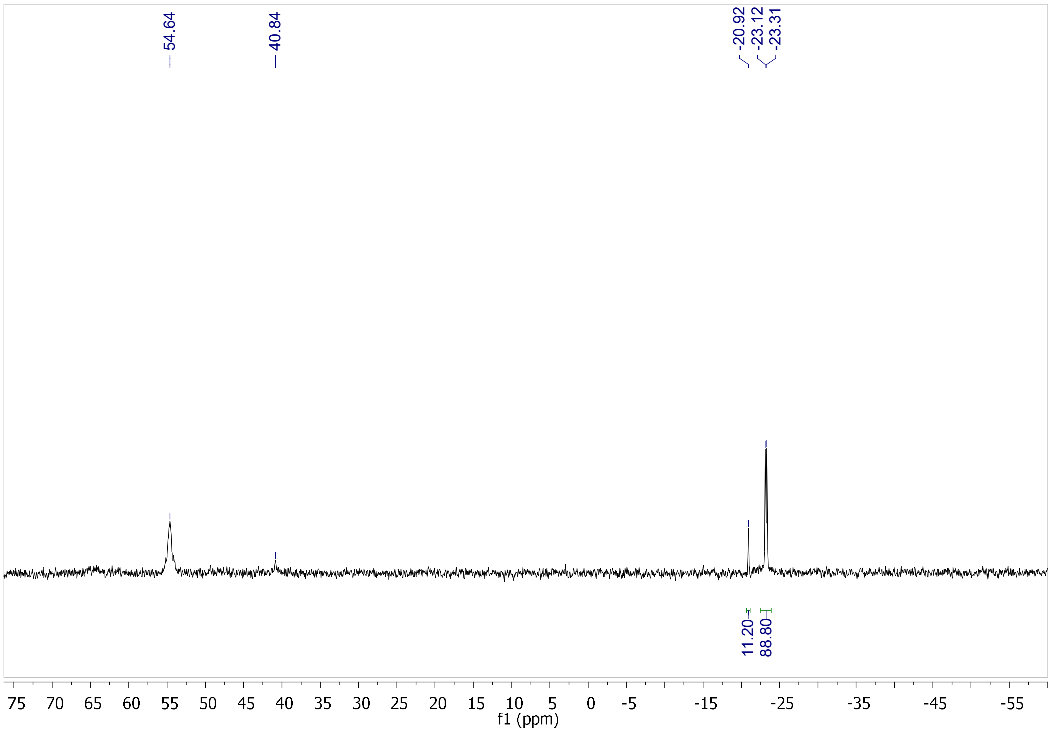

Figure S2d. ^{31}P-NMR of *mer*-**2** ($CDCl_3$).

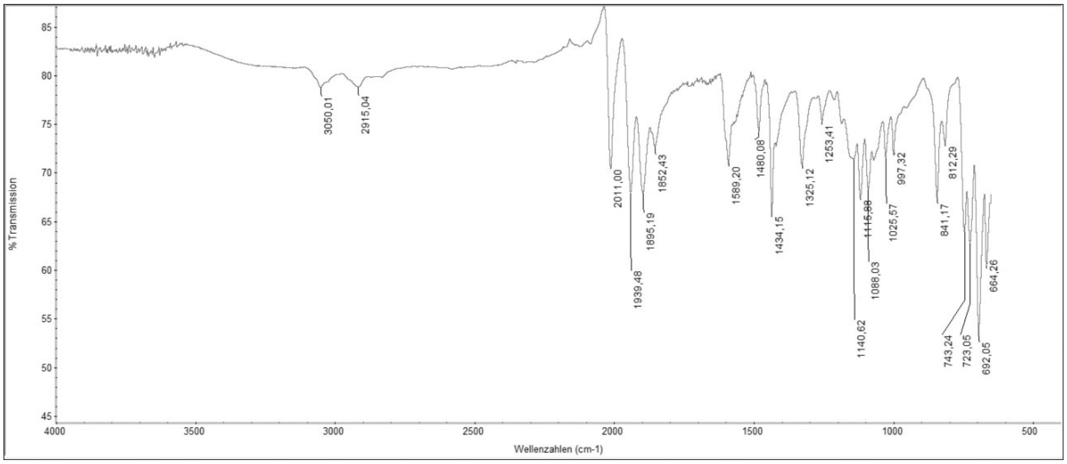

Figure S2e. IR spectrum of *mer*-**2**.

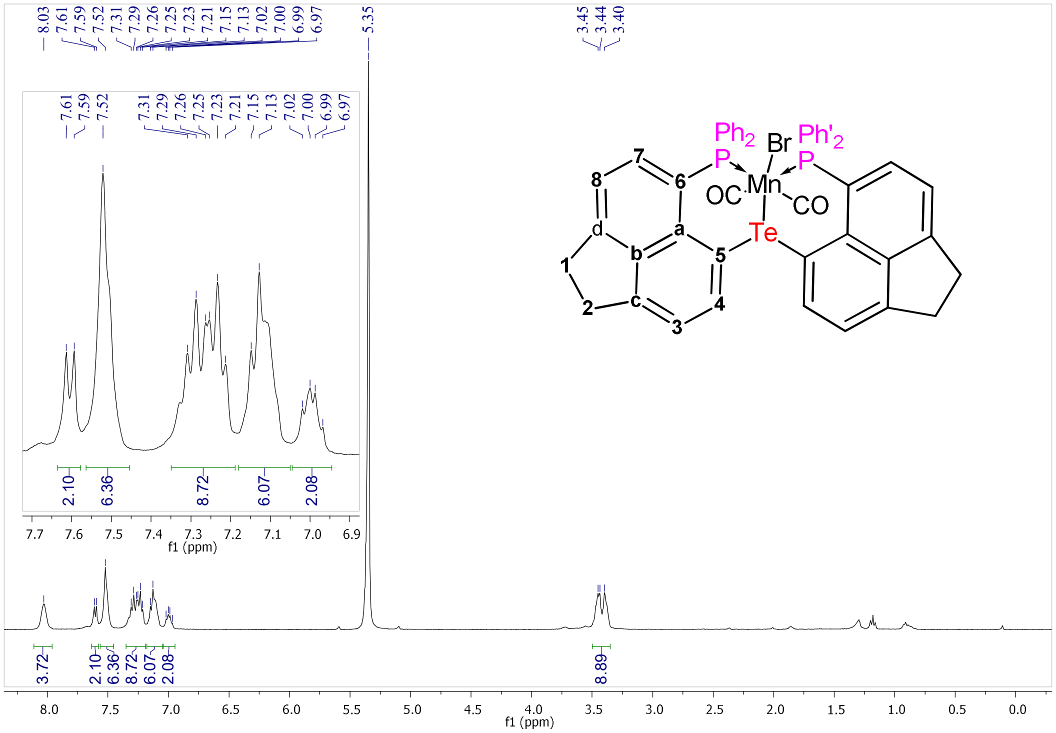

Figure S3a. ^{1}H-NMR spectrum of **3** (CD_2Cl_2).

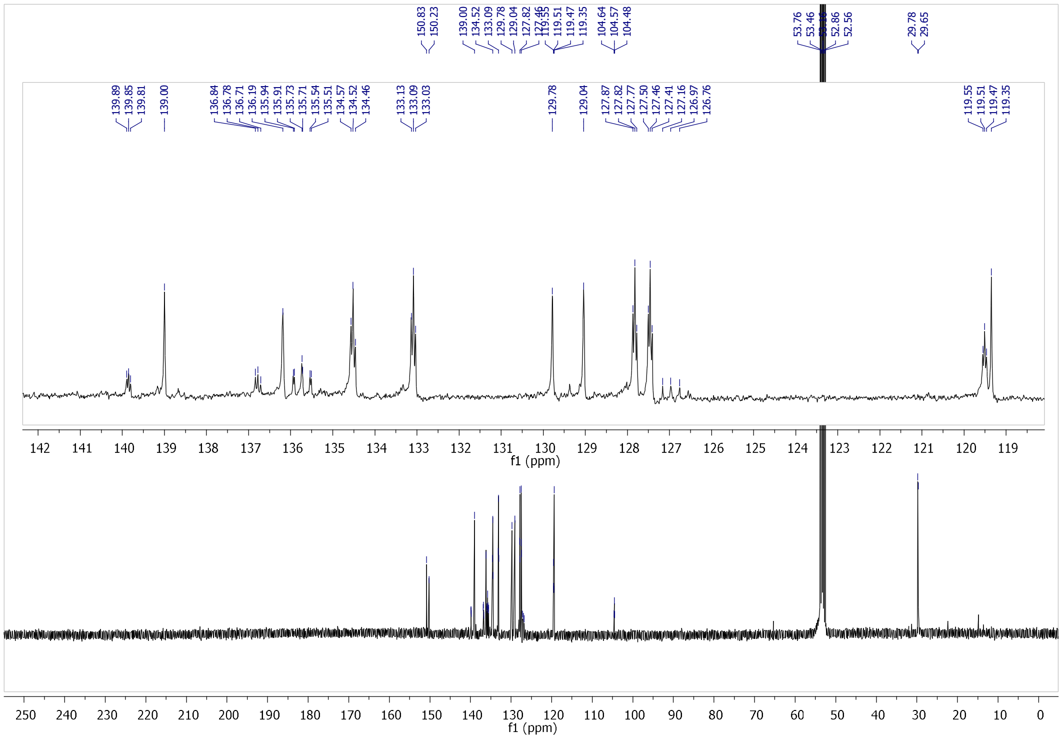

Figure S3b. $^{13}C\{^{1}H\}$-NMR spectrum of **3** (CD_2Cl_2).

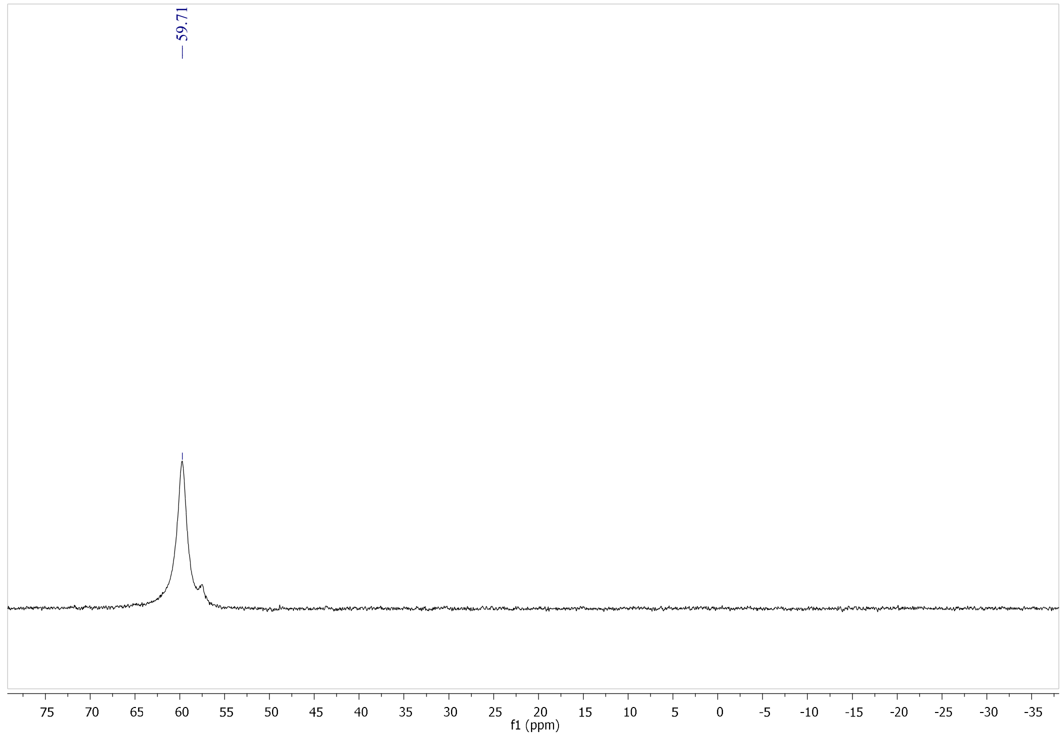

Figure S3c. $^{31}P\{^1H\}$-NMR spectrum of **3** (CD_2Cl_2).

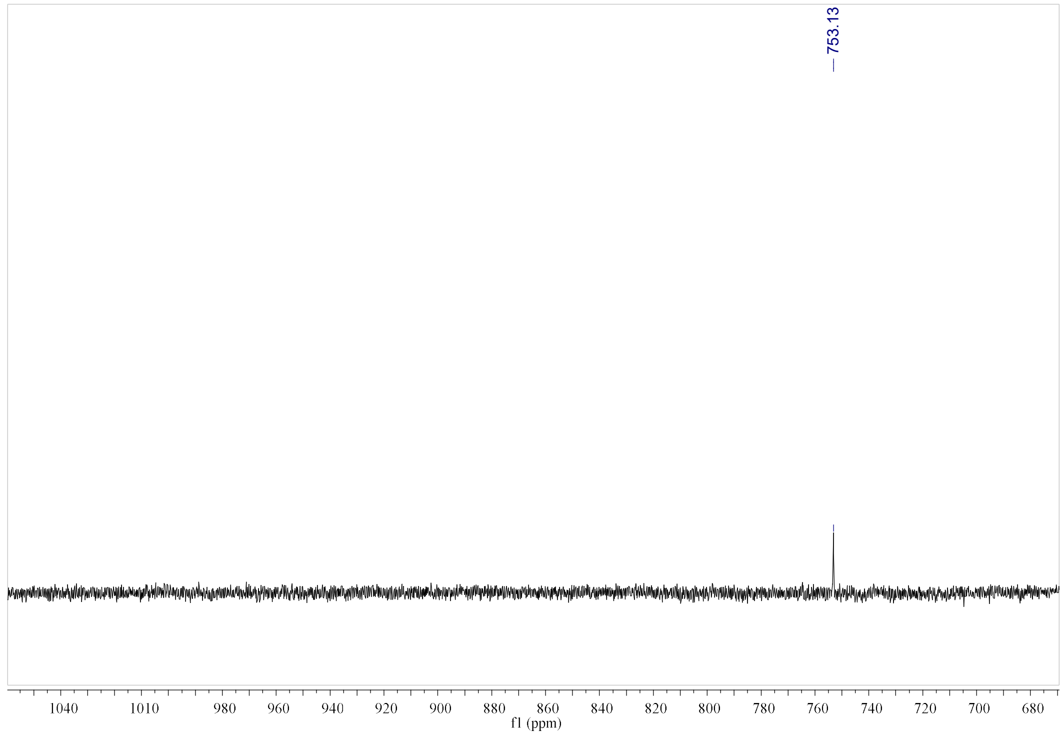

Figure S3d. $^{125}Te\{^1H\}$-NMR spectrum of **3** (CD_2Cl_2).

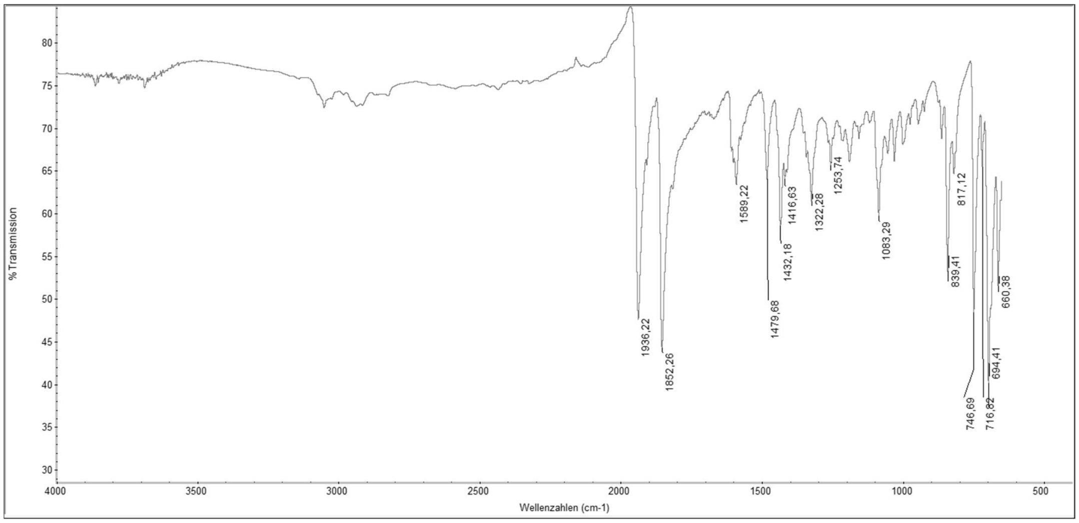

Figure S3e. IR spectrum of **3**.

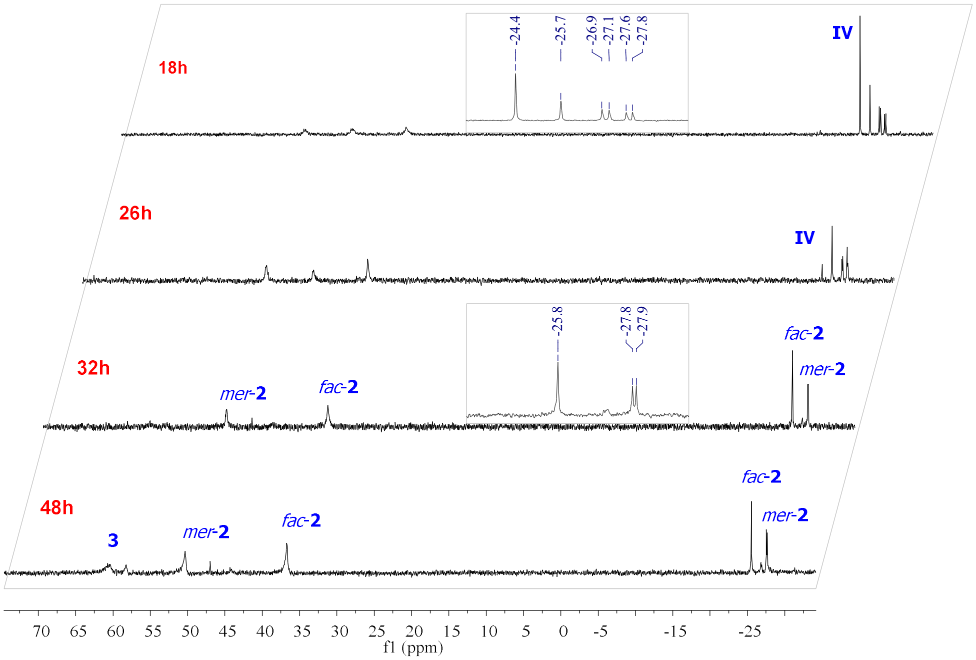

Figure S4a. Stacked plot of the $^{31}P\{^1H\}$ NMR spectra (THF) showing formation and the equilibrium between *fac*-**2**, *mer*-**2** and the formation of **3**.
